# A Study on the Electro-Optical Properties of Thiol-Ene Polymer Dispersed Cholesteric Liquid Crystal (PDChLC) Films

**DOI:** 10.3390/molecules22020317

**Published:** 2017-02-22

**Authors:** Yujian Sun, Yanzi Gao, Le Zhou, Jianhua Huang, Hua Fang, Haipeng Ma, Yi Zhang, Jie Yang, Ping Song, Cuihong Zhang, Lanying Zhang, Fasheng Li, Yuzhen Zhao, Kexuan Li

**Affiliations:** 1 Department of Materials Physics and Chemistry, School of Materials Science and Engineering, University of Science and Technology Beijing, Beijing 100083, China; syj_anshan@sina.com (Y.S.); jianhua_ustb@163.com (J.H.); Fanghua0229@126.com (H.F.); 5896326123@163.com (Y.Z.); g20158323@xs.ustb.edu.cn (J.Y.); 2 Department of Materials Science and Engineering, College of Engineering, Peking University, Beijing 100871, China; luoqiudehudie@163.com (L.Z.); mahaipeng6666@163.com (H.M.); songping1985@163.com (P.S.); 3 Key Laboratory of Polymer Chemistry and Physics of Ministry of Education, Peking University, Beijing 100871, China; 4 College of Medical Laboratory, Dalian Medical University, Dalian 116044, Liaoning, China; 5 Department of Applied Statistics and Science, Xijing University, Xi’an 710123, Shaanxi, China; 6 Anshan Normal University, Anshan 114005, Liaoning, China

**Keywords:** polymer dispersed cholesteric liquid crystal, liquid crystal, chiral, electro-optical performance

## Abstract

In this study, a polymer dispersed cholesteric liquid crystal (PDChLC) film obtained via a one-step fabrication technique based on photopolymerization of a thiol-acrylate reaction system was prepared and characterized for the first time. The effects of the chiral dopant, the influence of thiol monomer functionality and content on the morphology and subsequent performance of the PDChLC films were systematically investigated. It was demonstrated that the addition of a small amount of chiral dopant slightly increased the driving voltage, but decreased the off-state transmittance significantly. Furthermore, scanning electron micrographs (SEM) shown that the liquid crystal (LC) droplet size decreased at first and then increased with the increasing amount of thiol monomer functionality, while increasing the thiol content increased the LC droplet size. Correspondingly, the electro-optical switching behavior was directly dependent on LC droplet size. By tuning the raw material composition, PDChLC film with optimized electro-optical performance was prepared.

## 1. Introduction

Polymer dispersed liquid crystal (PDLC) films are a kind of functional macromolecular material, which consist of liquid crystal droplets dispersed in a semi-continuous polymer matrix [1,2,3]. Generally, a PDLC film shows electro-optical properties in a normal mode, which exhibits a light scattering and a transparent state without and with the application of an electric field, respectively. The normal scattering state (off-state) is due to the randomly oriented liquid crystal (LC) droplets dispersed in the polymer matrix, and the transparent state (on-state) is attributed to the parallel alignment of the LC directors with the applied electric field if there is a refractive index match between the LC droplets and the polymer matrix. These optical properties are valuable for optical shutters, display devices, smart windows and other devices [4,5,6,7], and combinations with other materials could expand the scope of application [8,9]. Nanostructures based on LCs are also getting more and more attention [10,11,12].

Four methods have been used to prepare PDLC films, including the encapsulation process (MP), thermally induced phase separation (TIPS), solvent-induced phase separation (SIPS) and polymerization-induced phase separation (PIPS) [13,14,15]. PIPS is considered the most popular method since the fabrication process is relatively simple, clean and solvent-free. Compared with the thermal curing method which takes several hours at least, the UV-initiated polymerization method is more efficient and controllable. At present, researchers are focusing more on the photo-polymerization-induced phase separation method based on acrylate and methacrylate monomers [16]. It is well known that the polymer precursor composition influences the performance characteristics of PDLC films such as transmittance and switching voltage. For example, introducing fluorinated acrylates into PDLCs can reduce the LC anchoring energy and subsequently lower the switching voltage [17]. In addition, a number of studies examining PDLCs have utilized the thiol-ene system consisting of a series of thiols with different functionality and urethane allyl ether as well as vinyl ether monomers [18,19]. Compared with the acrylate systems, the thiol-ene system has significant advantages, such as less light initiator dosage, excellent thermal insulation, high refractive index, inertia to oxidation and water resistance [20,21,22,23,24,25,26,27,28,29].

However, up till now, little attention has been paid to the use of acrylate monomer/thiol monomer for ultraviolet (UV)-initiated polymerization. In this study, a polymer dispersed cholesteric liquid crystal (PDChLC) film was prepared based on photopolymerization of the thiol-acrylate reaction system and characterized for the first time. The influences of the thiol functionality and the amount of thiol on the morphology and electro-optical properties of the resulting PDChLC films were carefully investigated.

## 2. Results and Discussions

### 2.1. The Effects of Different Amounts of Chiral Dopant on the PDChLC Films

#### 2.1.1. Morphology of the Polymer Network in PDChLC Films

**Figure 1:**
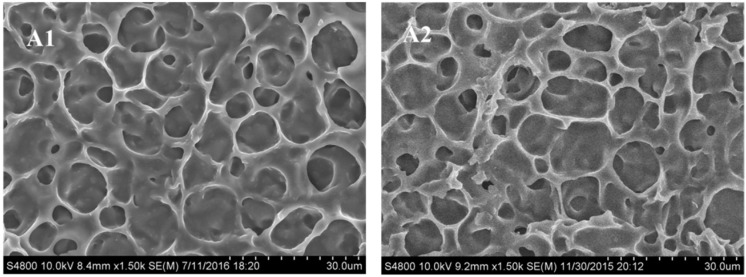

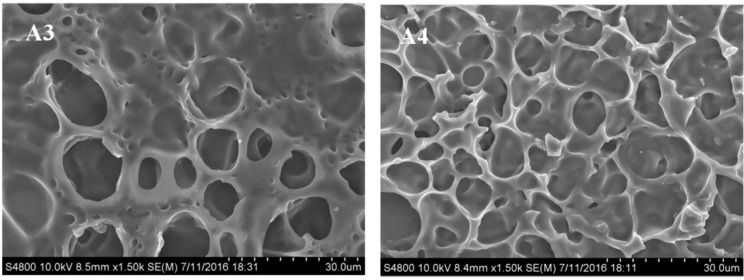
Scanning electron micrographs (SEM) of the polymer networks of the samples A1–A4.

Figure 1 shows scanning electron micrographs (SEM) micrographs of the polymer networks of samples A1–A4. As can be seen the LC domain size was slightly decreased with increasing chiral dopant (S811) content. It is well known the droplet size is controlled by the relative content of the photopolymerizable monomers and LC, the rate of polymerization, and some physical parameters such as the viscosity, rate of diffusion, and solubility of the LC in the polymer. For a definite system, the relative content of photopolymerizable monomer and LC remains the same, so increasing the S811 content caused the pitch to decrease, the anchoring force from the polymer network on the liquid crystals to increase, and this resulted in a decrease in the LC droplet size, which was not obvious because of the low amount of chiral dopant used.

#### 2.1.2. Electro-Optical Properties of PDChLC Films

**Figure 2:**
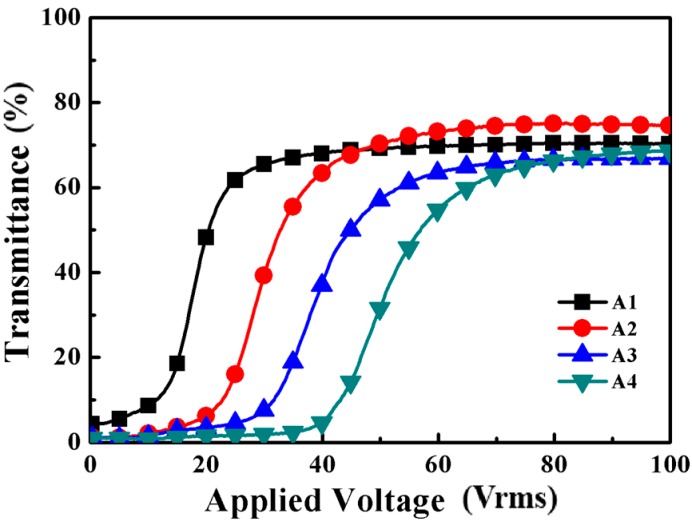
The transmittance-applied voltage curves of samples A1–A4.

The electro-optical properties of PDChLC films include driving voltages, response times and contrast ratio (CR) which are affected by the size, shape and anchoring energy of the LC droplets. The applied voltage-transmittance curves of samples A1–A4 are shown in Figure 2.

**Figure 3:**
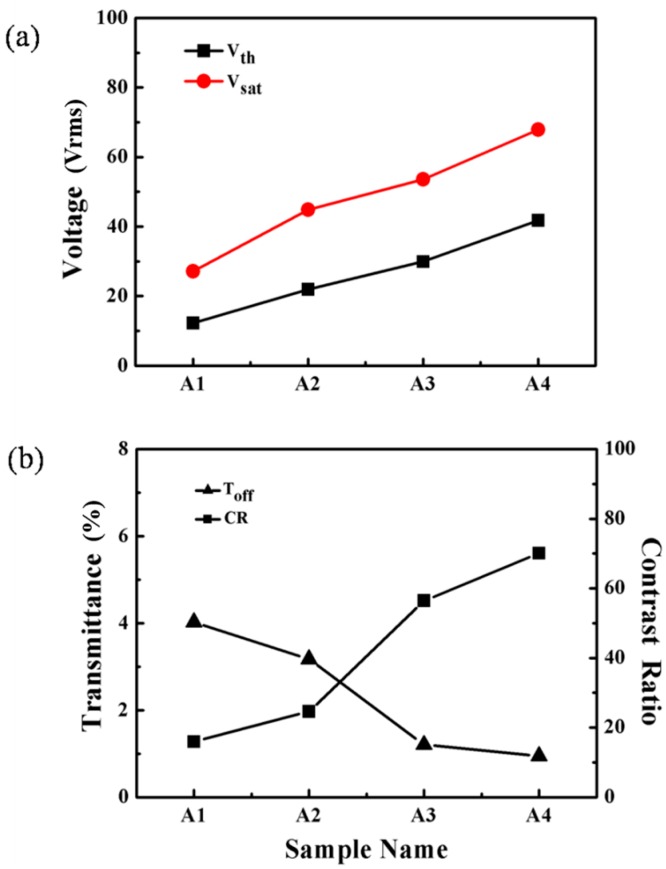
(**a**) Threshold voltage (*Vth*) and saturation voltage (*Vsat*) of samples A1–A4; (**b**) Off-state light transmittance (Toff) and contrast ratio (CR) of samples A1–A4.

The threshold voltage (*Vth*) and the saturation voltage (*Vsat*) are defined as the electric voltage required for the transmittance to reach 10% and 90%, respectively. Generally, the threshold voltage *Vth* is a linear function of the reciprocal of the size of the droplets *R* according to:

(1) $$V_{th}=\frac{d}{R}{\left[\frac{K\left(\omega^{2}-1\right)}{\varepsilon_{0}\times \Delta \varepsilon }\right]}^{\frac{1}{2}}$$

where *d*, *K*, *ω* and *Δε* represent film thickness, elastic constant, aspect ratio and dielectric anisotropy of the LC, respectively [30,31,32,33]. *Vth* is in inverse proportion to the LC domain size. Figure 3a shows *Vth* and *Vsat* of samples A1–A4, with the increase of S811 in contents, the *Vth* and the *Vsat* gradually increased from 15.9 V to 34.6 V and from 35.1 V to 58.1 V, respectively. It is possible to observe a change in the *Vth* and the *Vsat* whose increase could decrease the LC domain size. It is well known that for a defined system the increasing content of S811 could cause the pitch to decrease and the viscosity to increase, so the anchoring force from the polymer network on liquid crystals increases, which results in a decreasing of the LC droplet size. Therefore, *Vth* and *Vsat* of the PDLC films increased with the increasing content of S811.

The CR is an important measure of the electro-optical performance in a PDChLC system. The contrast ratio (CR, T_on_/T_off_) is commonly known as the switching contrast ratio, where T_on_ and T_off_ are the ultimate on-state transmittance and the initial off-state transmittance respectively. It is well-known that T_off_ of PDChLC films depends greatly on LC domain size, and appropriate LC domain sizes could provide sufficient scattering centers. As shown in Figure 3b, the CR of the samples A1–A4 increased with the increasing content of the S811.

It is well-known that the CR of PDChLC films depends greatly on LC domain size, so the CR increases with the decreasing LC droplet size. It can be seen that the sample A2 had a slightly lower contrast ratio, but it had a good polymer network leading to a high on-state transmittance and low *Vth*, which are of great importance for the performance of the films. Hence, the optimum electro-optical properties of the PDLC films were obtained when the content of the chiral dopant was 3 wt %.

### 2.2. The Thiol Functionality Effects in PDChLC Films

#### 2.2.1. Morphology of the Polymer Network in PDChLC Films

**Figure 4:**
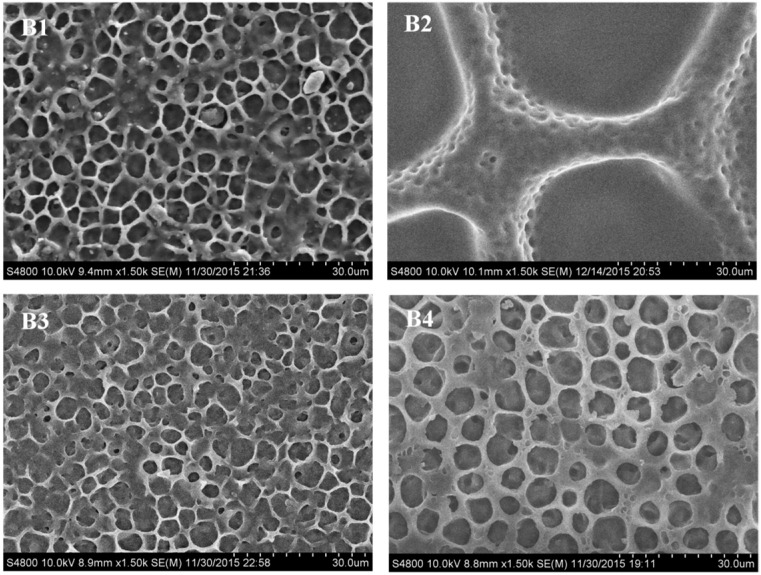
SEM micrographs of the polymer networks of the samples B1–B4.

Figure 4 shows the SEM micrographs of the polymer networks of samples B1–B4. Compared with sample B1, samples B2–B4 introduced co-polymerized thiol monomer into the compositions.

As a result of the reactivity of thiol monomer which only forms C–S bond though “thiol-ene” polymerization, the crosslinking density of acrylate monomers would be deceased due to the introduction of thiol monomer, so there were larger and more inhomogeneous domains, which may result from competition between thiol-acrylate reactions and acrylate monomer polymerization reactions. It can be seen that with the increase of thiol monomer functionality, the mesh size initially presented a decreasing trend, followed in sequence by an increase (as shown in samples B2–B4), which is related to the crosslinking density. Due to the linear molecular structure of 2,2′-(ethylenedioxy)diethanethiol (DET), the crosslinking density of sample B2 is lower than that of sample B3, leading to a bigger LC domain in sample B2. As for changes in the thiol monomer functionality, the LC droplet size increased with thiol monomer change from trimethylopropane tris(3-mercaptopropionate) (TTMP) to pentaerythritol tetra(3-mercaptopropionate) (PETMP), which may be due to the steric hindrance of the PETMP monomer that hinders the internal rotation of the molecular chain. The LC domain size increased with decreasing polymerization rate. The results suggest that it is possible to regulate the LC domain size by adjusting the functionality of the co-polymerized thiol monomer.

#### 2.2.2. Electro-Optical Properties of PDChLC Films

**Figure 5:**
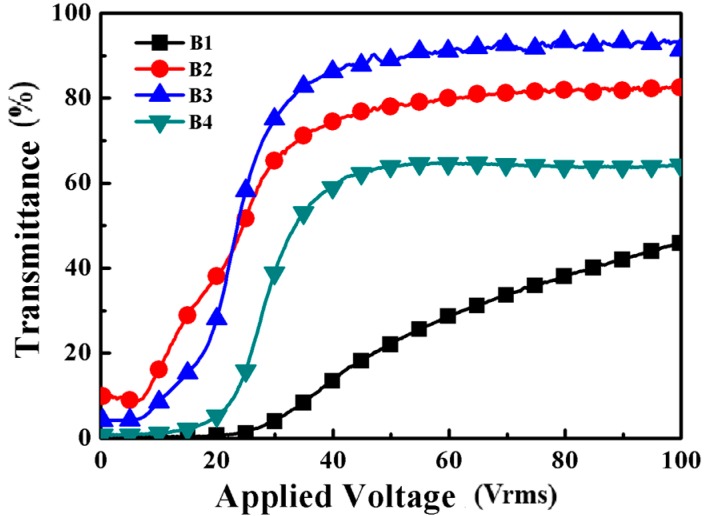
The transmittance-applied voltage curves of samples B1–B4.

**Figure 6:**
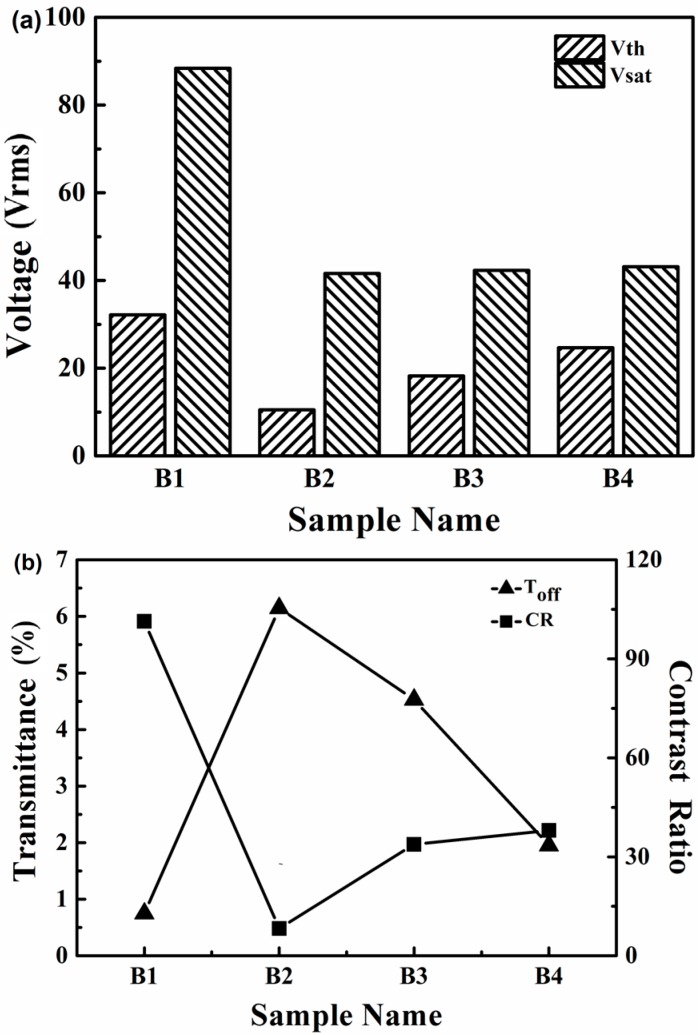
(**a**) *Vth* and *Vsat* of samples B1–B4; (**b**) Toff and CR of samples B1–B4.

Electro-optical properties are very important in the evaluation of PDLC films. As shown in Figure 5, the *Vth* and *Vsat* of sample B1 which was prepared without thiol were obviously higher than the others. The results show that it was an efficient method to reduce the driving voltage of PDChLC films by introducing co-polymerized thiol monomer. With the increase of the thiol monomer functionality of sample B2 to sample B4, the *Vth* increased gradually, while the *Vsat* fluctuated a little. The *Vth* of sample B3 was higher than that of sample B2 resulted from the decreasing size of the LC droplets. On the contrary, the *Vth* of sample B3 was smaller than that of sample B4, maybe due to a much more homogeneous porous structure. Therefore, the size and shape of the LC droplets synergistically affect the electro-optical performance of the film. The *Vth* and *Vsat* values of the samples were summarized in Figure 6a. Figure 6b shows the off-state light transmittance and the CR of samples B1–B4. It can be seen the sample B1 without thiol had the highest CR value due to the strong light scattering from smaller LC droplets. However, the low on-state transmittance limits its practical application. With the increase of the thiol functionality, the T_off_ of the samples B2–B4 decreased dramatically from 6.1% to 2.0%, while the T_on_ initially increased followed by a slight decrease. The CR of sample B4 is up to 85.6. However, the on-state transmittance of sample B4 was not very good, so subsequent studies were conducted to investigate the effect of different amounts of PETMP on the properties of the films.

### 2.3. The Effects of Different Amounts of Thiol on the PDChLC Films

#### 2.3.1. Morphology of the Polymer Network in PDChLC Films

**Figure 7:**
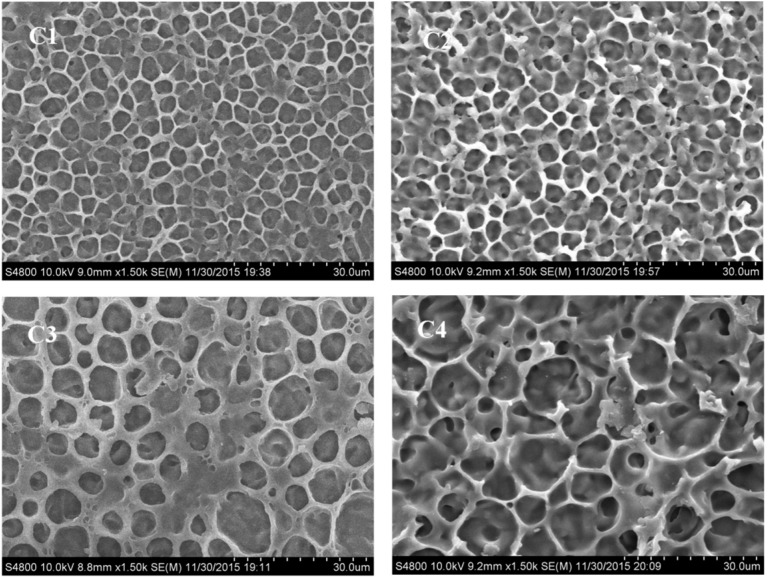
SEM micrographs of the polymer network in the samples C1–C4.

From Figure 7, it can be seen that as the ratio of monomer PETMP increased from 1 wt % to 7 wt %, the number of LC domains decreased and the domain sized increased. This phenomenon may be due to the competitive reaction between the thiol-acrylate reaction and acrylate monomer polymerization reaction. However, the inhomogeneous LC domains made the electro-optical properties of the PDChLC films complicated, and it was necessary to conduct further study of the electro-optical properties.

#### 2.3.2. Electro-Optical Properties of PDChLC Films

**Figure 8:**
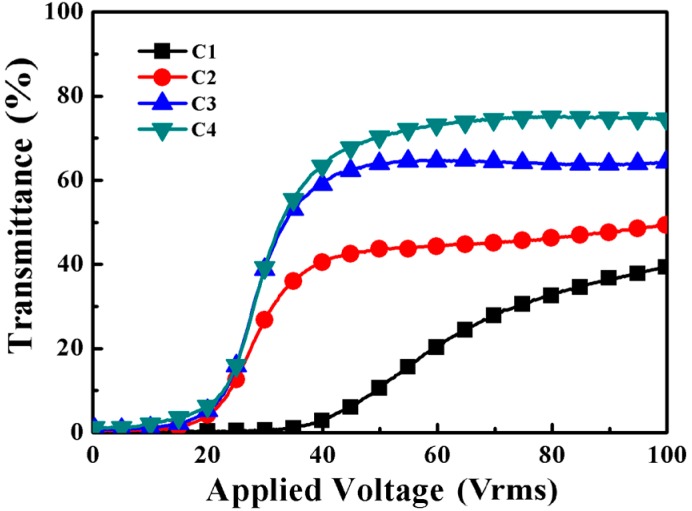
The transmittance-applied voltage curves of samples C1–C4.

**Figure 9:**
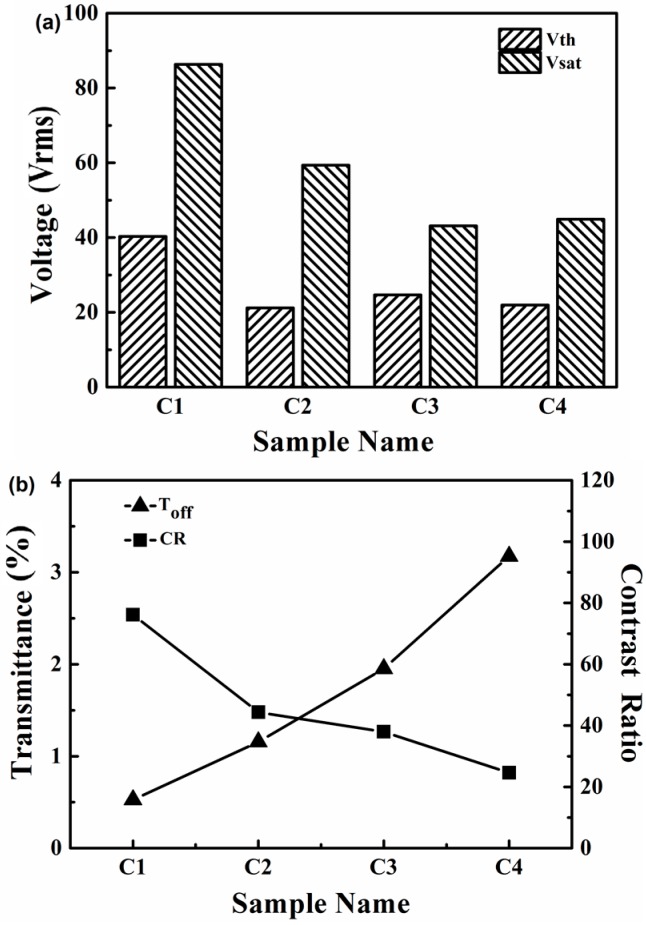
(**a**) *Vth* and *Vsat* of samples C1–C4; (**b**) Toff and CR of samples C1–C4.

The electro-optical properties of samples C1–C4 are shown in Figure 8. Sample C4 prepared with 7 wt % PETMP presented better properties than the others. According to Equation (1), the electro-optical properties of PDLC films can be controlled by the LC domain size and some other factors, such as film thickness, the resistivity and the dielectric anisotropy of the LC. Therefore, *Vth* and *Vsat* of the PDLC films decreased with the increasing LC domain size. For C1, the domains were smaller with higher *Vth* and *Vsat*, and its on-state transmittance was too low to be taken into consideration, although its contrast ratio was high. Secondly, the LC domain size and distribution variations were also related to T_on_ and T_off_, as shown in Figure 9. As the LC domains became larger, the anchoring energy decreased and this led the T_off_ of the samples C1–C4 to increase from 0.5% to 3.2%, while the T_on_ increased dramatically from about 40.2% to 74.4%, as shown in Figure 9. Compared to C2 and C3, sample C4 with 7 wt % PETMP had a slightly lower contrast ratio, but it had a good polymer network leading to a high on-state transmittance, which was of great importance for the performance of the films. Hence, we can reach the conclusion that adding 7 wt % PETMP as the co-polymerized monomer could result in a “thiol-acrylate” PDChLC film with optimized electro-optical performance.

## 3. Materials and Methods

### 3.1. Materials

**Figure 10:**
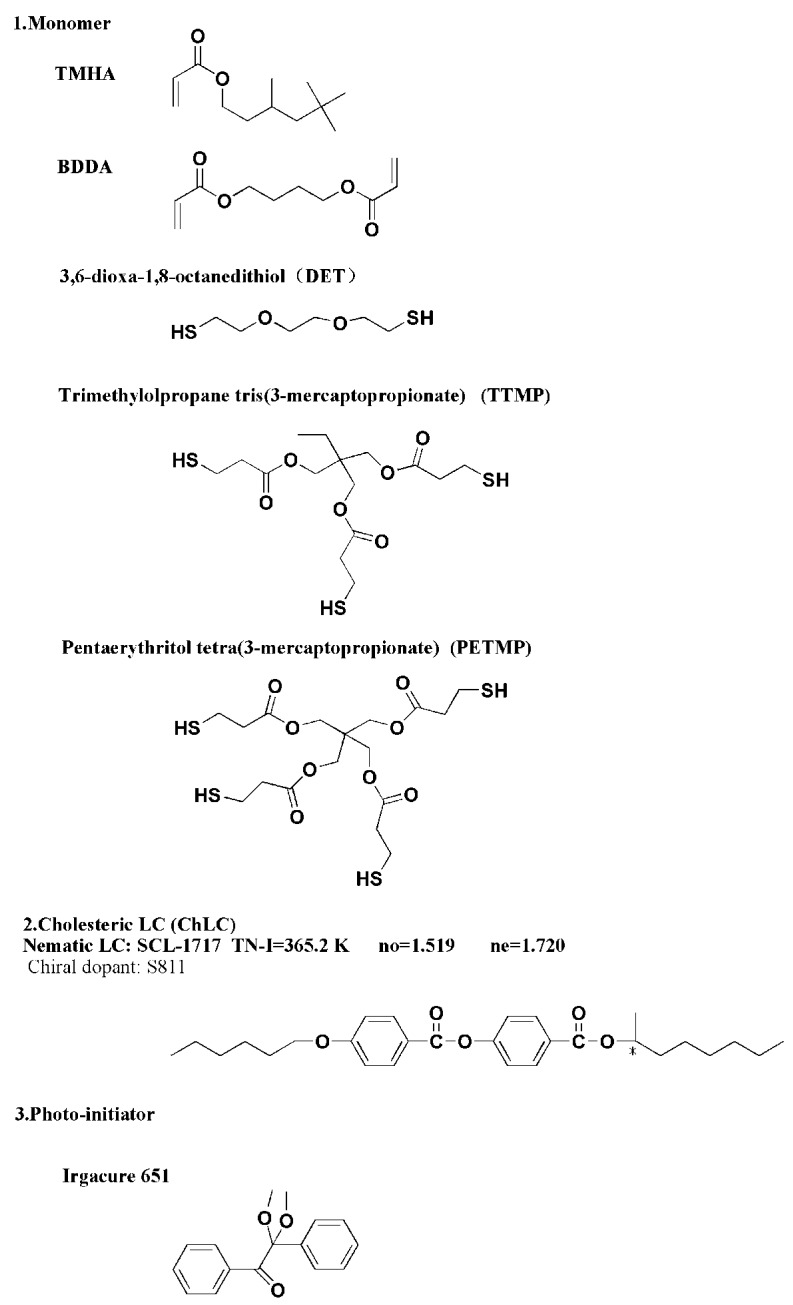
The chemical structures of the materials used. TMHA: 3,5,5-Trimethylhexylacrylate; BDDA: 1,4-butanedioldiacrylate; LC: liquid crystal; SCL-1717: nematic liquid crystal; no: ordinary refraction index; ne: extraordinary refraction index.

The cholesteric liquid crystal (ChLC) used consisted of a nematic LC (SLC-1717, Shijiazhuang Chengzhi Yonghua Display Material Co., Ltd., Shijiazhuang, China) and a chiral dopant (S811, Merck Co., Ltd., Kenilworth, NJ, USA). The acrylate monomer was a mixture of 3,5,5-trimethelhexyl acrylate (TMHA, 98%, Sigma-Aldrich, St. Louise, MO, USA) and 1,4-butanedioldiacrylate (BDDA, 98%, Tokyo Chemical Industry Co., Ltd., Tokyo, Japan). The mercaptan monomers were 2,2′-(ethylenedioxy)diethanethiol (95%), trimethylopropane tris(3-mercaptopropionate) (95%) and pentaerythritol tetra(3-mercaptopropionate) (95%) which were purchased from J&K Scientific Ltd. (Chaoyang District Beijing, China). The photoinitiator was Irgacure 651 (Ciba Geigy, Jingjiang Hongtai Chem. Co., Ltd., Jingjiang, China). All these materials were used without further purification and the structures are shown in Figure 10.

### 3.2. Sample Preparation

The prepared samples consisted of acrylate monomers, thiol, photo-initiator and a constant amount of ChLC (80.0 wt %). Firstly, they were thoroughly mixed in the specified proportions until a homogeneous mixture was formed. Then, based on capillary action, the mixture was filled into the LC cell whose inner faces had been coated with indium tin oxide (ITO). The film thickness was controlled by a 20.0 ± 1.0 μm polyethylene terephthalate (PET) spacer. After this, the samples were irradiated for polymerization by a UV lamp (365 nm, 35 W Hg lamp, PS135, UV Flood, Stockholm, Sweden) for 5 min at 25 °C, and the curing intensities at the cell surface were about13.5 mW/cm^2^ at 365.0 nm. The compositions of the samples are listed in Table 1.

### 3.3. Measurements

Scanning electron microscopy (S-4800, Hitachi, Tokyo, Japan) was used to observe the morphologies of the PDChLC films. The films were first cut into small pieces and then soaked in hexane for 9 days at room temperature to extract the LC from the polymer matrix. After that, the samples were dried under vacuum for 10 h. Then the surface of the polymer matrix was sputtered with gold and the microstructure of the polymer network was observed by SEM. A liquid crystal device (LCD) parameter tester (LCT-5016C, Changchun Liancheng Instrument Co., Ltd., Changchun, China) was used to measure the electro-optical properties of the samples at room temperature. A halogen laser beam (λ = 560 nm) was used as the incident light source. The transmittance of the PDChLC films was recorded by a photodiode, which was further monitored by a digital storage oscilloscope. An electric field square wave (100 Hz) was applied and the distance between the PDChLC film and photodiode was 300 mm. The collection angle of the transmitted intensity was about ±1° so that the forward scattering was mainly detected.

## 4. Conclusions

In summary, PDChLC films based on thiol/acrylate monomers were prepared and characterized. The morphology of the PDChLC film was strongly influenced by the chiral dopant and the addition of different amounts of S811 along the network bone affected the LC domain size distribution, which in turn influenced the electro-optical properties of the PDChLC films. Furthermore, the effect of the co-polymerized thiol monomer functionality and its content in the PDChLC films has been studied. As mentioned above, the PDChLC film prepared with 7 wt % PETMP to which 3 wt % S811 was added had better electro-optical properties than that prepared with other amounts of PETMP. The results in this study suggest that the chiral dopant plays an important role in the formation of the polymer network, and it is possible to regulate the LC domain size and optimize the electro-optic properties of PDLC films by adjusting the content of S811 and the thiol/acrylate feed ratio.

## Figures and Tables

**Table 1:** The compositions of all the samples.

Sample	Monomers		SLC1717/S811 (wt %)
	Composition of Monomers	Weight Ratio	
**Group A**			
A1	TMHA/BDDA/PETMP	10.4/2.6/7.0	78.0/2.0
A2	TMHA/BDDA/PETMP	10.4/2.6/7.0	77.0/3.0
A3	TMHA/BDDA/PETMP	10.4/2.6/7.0	76.0/4.0
A4	TMHA/BDDA/PETMP	10.4/2.6/7.0	75.0/5.0
**Group B**			
B1	TMHA/BDDA	16.0/4.0	77.0/3.0
B2	TMHA/BDDA/DET	12.0/3.0/5.0	77.0/3/0
B3	TMHA/BDDA/TTMP	12.0/3.0/5.0	77.0/3.0
B4	TMHA/BDDA/PETMP	12.0/3.0/5.0	77.0/3.0
**Group C**			
C1	TMHA/BDDA/PETMP	15.2/3.8/1.0	77.0/3.0
C2	TMHA/BDDA/PETMP	13.6/3.4/3.0	77.0/3.0
C3	TMHA/BDDA/PETMP	12.0/3.0/5.0	77.0/3.0
C4	TMHA/BDDA/PETMP	10.4/2.6/7.0	77.0/3.0

DET: 2,2′-(ethylenedioxy)diethanethiol; TTMP: trimethylopropane tris(3-mercaptopropionate); PETMP: pentaerythritol tetra(3-mercaptopropionate); S811: chiral dopant.
